# The Link Between Newborn SNP Polymorphism rs266729, Adiponectin, and Newborn Macrosomia in a Cohort of Pregnant Women with Gestational Diabetes Mellitus: A Case–Control Pilot Study

**DOI:** 10.3390/children12020155

**Published:** 2025-01-28

**Authors:** Mihai Muntean, Claudiu Mărginean, Elena Silvia Bernad, Claudia Bănescu, Victoria Nyulas, Irina Elena Muntean, Vladut Săsăran

**Affiliations:** 1 Department of Obstetrics and Gynecology 2, George Emil Palade University of Medicine, Pharmacy, Science, and Technology of Târgu Mureș, 540142 Targu Mures, Romania; mihai.muntean@umfst.ro (M.M.); vladut.sasaran@umfst.ro (V.S.); 2 Department of Obstetrics and Gynecology, Faculty of Medicine, “Victor Babes” University of Medicine and Pharmacy, 300041 Timisoara, Romania; bernad.elena@umft.ro; 3 Clinic of Obstetrics and Gynecology, “Pius Brinzeu” County Clinical Emergency Hospital, 300723 Timisoara, Romania; 4 Center for Laparoscopy, Laparoscopic Surgery and In Vitro Fertilization, “Victor Babes” University of Medicine and Pharmacy, 300041 Timisoara, Romania; 5 Genetics Laboratory, Center for Advanced Medical and Pharmaceutical Research, George Emil Palade University of Medicine, Pharmacy, Science, and Technology of Târgu Mureș, 540142 Targu Mures, Romania; claudia.banescu@umfst.ro; 6 Department of Informatics and Medical Biostatistics, George Emil Palade University of Medicine, Pharmacy, Science, and Technology of Târgu Mureș, 540142 Targu Mures, Romania; victoria.rus@umfst.ro; 7 Algcocalm SRL, 540360 Targu Mures, Romania; irina.muntean@algocalm.ro

**Keywords:** gestational diabetes mellitus, macrosomia, adiponectin, gene, rs266729

## Abstract

Background: Gestational diabetes mellitus (GDM) is linked to higher newborn weight and an increased risk of macrosomia. The newborn single-nucleotide polymorphism (SNP) of the ADIPOQ gene rs266729 is linked to a higher birth weight of the offspring of healthy pregnant women. Objectives: This study aims to evaluate the relationship between newborn ADIPOQ rs266729 polymorphism, cord blood adiponectin, maternal glycemic and lipid metabolism, and maternal adiponectin levels at 24 to 28 weeks of gestation (WG) and at birth and its impact on newborn weight in a cohort of GDM mothers. Materials and methods: This study involved 71 women diagnosed with GDM and 142 control pregnant women. The ADIPOQ (rs266729) gene polymorphisms were genotyped using TaqMan real-time PCR analysis. Maternal and cord blood adiponectin levels were measured using human total adiponectin ELISA kits. We performed a Pearson correlation analysis to identify significant correlations between maternal metabolic parameters and adiponectin levels at 24–28 WG and birth and the weight of newborns. A logistic regression analysis was also conducted to identify potential macrosomia predictors. Results: We found no significant differences in the distribution of the allele (C, G) (*p* = 0.82) and genotype (CC, CG, GG) (*p* = 0.46) of APIPOQ rs266729 among normoponderal and macrosomic newborns from the GDM mothers group. Maternal fasting glucose at 24–28 WG was higher in the GDM mothers who gave birth to macrosomic newborns (106 ± 17 vs. 93 ± 10 mg/dL, *p* < 0.0001). Adiponectin levels in the cord blood of newborns from mothers with GDM were lower than those in newborns from control mothers (*p* < 0.0001). In correlation analysis, we identified a weak positive correlation between the newborn weight of GDM mothers and cord blood adiponectin (r = 0.262), maternal fasting glucose level at 24–28 WG (r = 0.288), and maternal adiponectin level at birth (0.334). Multivariate logistic regression, after adjusting for confounders, revealed that maternal fasting glucose levels at 24–28 WG had an OR of 11.59, and cord blood adiponectin levels had an OR of 30.31 for macrosomia. Conclusions: The preliminary findings of our pilot study suggest that in the gestational diabetes mellitus group, the ADIPOQ rs266729 polymorphism in newborns is not associated with a higher birth weight, maternal fasting glucose levels between 24 and 28 WG were a predictor for macrosomia, and cord blood adiponectin levels were lower than those from control mothers. Further large-scale studies are needed to confirm our findings.

## 1. Introduction

The incidence of childhood and adolescent obesity is increasing worldwide [1,2], exposing young women to the risks associated with pregnancy in obese patients. Obesity associated with pregnancy predisposes the mother–child couple to complications in the short and long term [3]. Obese pregnant women have an increased risk for short-term complications like spontaneous abortion and congenital malformations [3], and due to increased insulin resistance, hyperglycemia, hyperinsulinemia, and lifestyle modification (high caloric intake and sedentarism), they have a high incidence of gestational diabetes mellitus (GDM) and hypertensive disorders of pregnancy [3,4,5]. GDM, the most prevalent metabolic complication of pregnancy, refers to diabetes diagnosed in the second or third trimester that was not overt diabetes before gestation, and it does not include other types of diabetes that can occur during pregnancy, such as type 1 diabetes (T1DM) [6].

The newborns of overweight and obese pregnant women are significantly heavier than those of normal-weight women [3,7]. Macrosomia is defined as a newborn with a birth weight (BW) > 4000 g [8]. Macrosomic newborns of GDM mothers have higher body fat levels than infants born to women with a normal glucose tolerance [9]. Macrosomia is associated with an increased risk of maternal complications (emergency cesarean section, postpartum hemorrhage, and obstetric anal sphincter injury) and neonatal complications (shoulder dystocia, obstetric brachial plexus injury, birth fractures, low APGAR score, and increased rate of admission to a neonatal intensive care unit) [8,10,11,12].

The long-term complications of maternal obesity for the mother and their newborn are an increased risk for obesity throughout life, metabolic syndrome, hypertension, and type 2 diabetes mellitus (T2DM) [8,10]. These complications arise through a process of fetal programming that includes various epigenetic, cellular, physiological, and metabolic mechanisms in an abnormal in utero environment [7,13,14].

Adipose tissue is recognized as a metabolically active endocrine organ that produces hormones known as adipokines [15]. One adipokine is adiponectin, which is found in multiple multimeric complexes and has insulin-sensitizing, anti-atherogenic, and anti-inflammatory properties [16]. It is encoded by the ADIPOQ gene on chromosome 3q27 [16].

Adiponectin levels in pregnant women with GDM are lower than in healthy pregnant women [17] and in macrosomic babies born to GDM mothers compared with age-matched control newborns [18]. One mechanism that could be implicated in fetal overgrowth in pregnant women with GDM is that low levels of maternal adiponectin, combined with increased levels of insulin growth factor 1 (IGF-1), could cause an increased expression of glucose transporter protein 1 (GLUT-1) via heightened activation of insulin/IGF-1 signaling in the placenta of these women. This results in an increased supply of nutrients to the fetus, contributing to fetal overgrowth [19].

Recent evidence shows that the G allele and genotype CG of rs266729 single-nucleotide polymorphisms (SNPs) of the ADIPOQ gene increase the risk for GDM in the European population [20], and others have shown that the C allele of rs266729 is associated with lower adiponectin levels in GDM patients [21]. Others have also shown that newborn SNPs of the ADIPOQ gene, like rs266729, rs182052, and rs2241766, are associated with a greater birth weight in healthy pregnant women [22,23] and that newborns with genotype GG of rs266729 have higher adiponectin levels than those with CC or CG genotypes [22]. All these data suggest that the influence of the rs266729 SNP in ADIPOQ on birth weight may depend on circulating adiponectin.

Therefore, there is a gap in knowledge regarding the influence of the rs266729 SNP on newborn weight and adiponectin levels in infants born to GDM mothers. Our study is unique in that no other studies have evaluated the influence of the rs266729 polymorphism on the birth weight of newborns of GDM mothers. This is important because it aims to provide more information regarding the complex physiopathology of this disease, which induces significant complications for the mother–newborn dyad.

Thus, the primary objectives of this study were to evaluate the effect of the newborn ADIPOQ rs266729 polymorphism on newborn weight status, to assess the impact of maternal glycemic and lipid metabolism and maternal adiponectin levels at 24 to 28 weeks of gestation and at birth on newborn weight, and to examine the connection between cord blood adiponectin and newborn weight in a cohort of pregnant women with GDM and their newborns.

## 2. Material and Method

### 2.1. Study Design

The University of Medicine, Pharmacy, Science and Technology “G. E. Palade” of Târgu-Mures Ethics Committee has authorized this study (decision number 1557/2022) following the principles of the Declaration of Helsinki (1964).

### 2.2. Description of Study Area and Duration of Study

We conducted a nested prospective case–control study, with an according-to-protocol analysis study design, from February 2022 to August 2024 at the Obstetrics–Gynecology Clinic 2 Unit of County Hospital Mureș in Târgu-Mureș, Romania.

### 2.3. Inclusion and Exclusion Criteria

We conducted an a priori power analysis using the G Power Version 3.1.9.6 program from Faul et al. [24] to determine the number of patients needed for the study, utilizing data from Atègbo et al. [18]. Based on these data, we estimated a medium effect size of 0.5, assuming a two-tailed *t*-test with at least 95% power and an alpha level of 0.05. The minimum required total of 206 patients will ensure adequate power, with 137 in the control group and 69 in the GDM group. We estimated that 213 patients, divided into 142 control patients and 72 patients in the GDM group (in a 2:1 ratio), would be sufficient for our study to maintain adequate power.

The inclusion criteria were singleton pregnancy, diagnosis of gestational diabetes mellitus (GDM) at 24–28 weeks of pregnancy, Romanian ethnicity, age above 18 years, and delivery at the Obstetrics and Gynecology Clinic 2 in Târgu-Mureș. The exclusion criteria were patients with type 1 diabetes mellitus (T1DM) or type 2 diabetes mellitus (T2DM) diagnosed before pregnancy, GDM diagnosed before 24 weeks of pregnancy, pregnancies with chromosomal anomalies or fetal malformations, cases of intrauterine fetal death, chronic infections, autoimmune and inflammatory diseases, stressful life events (financial, traumatic, spousal, and emotional), neoplastic diseases, and those who lacked informed consent. Among the exclusion criteria, we included stressful life events since everyday life is filled with potential stressors that may induce an inflammatory state, which can elevate the incidence of GDM [4,25]. Our exclusion criteria are clear, specific, transparent, and relevant to the research questions. However, since our study is a pilot study, their robustness can be enhanced in future studies.

Before joining the study, all pregnant women provided written informed consent for themselves and their newborns.

Before conducting anthropometric, biochemical, and genetic analyses, evaluators were blinded to group assignments. Structured questionnaires were used to obtain demographics (maternal age, gestation, parity, and first-degree family history of T2DM) and medical and reproductive history.

In each instance, we determined the gestational age by referencing the date of the last menstrual period alongside a first-trimester ultrasound.

### 2.4. Diagnosis of Gestational Diabetes Mellitus

We used the International Association of Diabetes and Pregnancy Study Groups (IADPSG) criteria [26] to diagnose gestational diabetes mellitus (GDM). One or more abnormal glucose values above ≥92 mg/dL (≥5.2 mmol/L) fasting, 1 h ≥ 180 mg/dL (≥10 mmol/L), or 2 h ≥ 153 mg/dL (≥8.5 mmol/L) after 75 g glucose ingestion were used for diagnosis.

Patients diagnosed with GDM were advised to engage in moderate exercise for 30 min daily, follow a nutritional plan ranging from 1600 to 1800 kcal per day with 35–40% of calories from carbohydrates, and monitor their glycemic levels by performing three daily checks of both fasting and postprandial glucose for two weeks following our local protocol. The target glucose levels were established as fasting below 95 mg/dL and postprandial levels under 120 mg/dL, to be measured two hours after meals [27]. A diabetologist recommended insulin therapy at a dosage of 0.7–1.0 units/kg of body weight daily for women unable to control their blood sugar through exercise and diet. Pregnant women were to monitor their glucose levels until delivery, closely overseen by the diabetologist. Each pregnant woman attended appointments every two weeks or more often if required, as part of their standard prenatal care.

### 2.5. Anthropometric Measurements

We conducted a series of maternal anthropometric measurements at the study’s inclusion point, specifically at 24 to 28 weeks of gestation (WG), and during hospital admission prior to birth for all pregnant women participating in the study. These measurements included weight, height, body mass index (BMI), and total weight gain during the study period of pregnancy. We utilized the patient’s weight before pregnancy, as reported during the initial prenatal visit, to compute her pre-pregnancy BMI.

Using a wall-mounted tape measure, we measured the patient’s height in centimeters without shoes, rounding the value to the nearest centimeter.

To assess the patient’s weight (kg), we used a Beurer PS digital scale (Beurer GmbH, Ulm, Germany), accounting for clothing by subtracting 500 g.

The patient’s BMI was calculated by dividing their weight by the square of their height (kg/m^2^).

All newborns were weighed and measured for weight and length in the first hour after birth. Their weight was assessed using the U-Grow electronic baby scale, U001-BS (Guangzhou Berrcom Medical Device Co., Ltd., Guangzhou, China), while their length was recorded with a non-stretchable tape measure.

### 2.6. Biochemical Analyses

We collected maternal blood samples at admission in the study at 24–28 weeks’ gestation (WG) and upon hospital admission during the prepartum period. Shortly after collection, at 24 to 28 weeks, we measured the fasting glucose level, 1 h glucose level, and 2 h glucose level after administering 75 g of oral glucose, along with total cholesterol (TC), high-density lipoprotein cholesterol (HDL-C), low-density lipoprotein cholesterol (LDL-C), triglycerides (TG), and adiponectin levels. During the prepartum period, we evaluated the maternal fasting glucose level, total cholesterol (TC), high-density lipoprotein cholesterol (HDL-C), low-density lipoprotein cholesterol (LDL-C), triglycerides (TG), and adiponectin levels. 

The Atellica Solution CH 930 device (Siemens Healthcare GmbH, Eschborn, Germany) was utilized to evaluate blood glucose levels via spectrophotometry, while TC, HDL-C, LDL-C, and TG levels were measured using photometry.

To assess adiponectin levels in newborns, we collected blood from the umbilical cord immediately after birth.

To evaluate adiponectin levels, the blood samples were placed in a serum separator tube at room temperature for 30 min to enable the serum to clot. Subsequently, they were centrifuged at 6000 rpm for 4 min at room temperature. The serum was separated and stored at −20 °C until August 2024, ensuring it did not undergo freeze–thaw cycles before being assayed. We utilized the DYNEX DSX Automated ELISA System (DYNEX Technologies Inc., Chantilly, VA, USA), an automated enzyme immunoassay analyzer, to measure adiponectin levels using the Human Total Adiponectin/ACRP30 ELISA kits (PDRP 300, R&D Systems, Bio-techne, Minneapolis, MN, USA), following the manufacturer’s protocol. The intra-assay variation for adiponectin was <4.8%, while the inter-assay variation was <7.0%. The manufacturer indicates that the sensitivity of the assays for adiponectin is 0.246 ng/mL.

### 2.7. Genotyping Analysis

In the prepartum period, we simultaneously collected maternal blood for genotyping and biochemical analysis, and for the newborn, we collected blood from the umbilical cord via puncture immediately after birth.

Blood samples were collected in tubes containing ethylenediaminetetraacetic acid (EDTA) and stored at −20 °C until August 2024, ensuring it did not undergo any freeze–thaw cycles before being assayed. DNA was extracted using a PureLink™ kit (Invitrogen, Life Technologies Corp, Carlsbad, CA, USA).

We genotyped all samples by using the TaqMan genotyping methodology, TaqMan™ Fast Advanced Master Mix (ThermoScientific LSG, Waltham, MA, USA), and specific TaqMan^®^ pre-designed TaqMan^®^ SNP Genotyping Assays (Applied Biosystems, Foster City, CA, USA) to discriminate the ADIPOQ rs266729 (C_2412786_10).

Genotyping was performed on the 7500 Fast DX Real-Time polymerase chain reaction (PCR) system (Applied Biosystems, Foster City, CA, USA).

### 2.8. Statistical Analysis

Statistical analyses were performed using GraphPad Prism version 9.0 (GraphPad Software, Boston, MA, USA). Continuous variables were expressed as the mean ± standard deviation and median (IQR). Categorical variables were expressed as percentages. The Kolmogorov–Smirnov test was used to assess the normality of the data. We applied the Student’s *t*-test for normally distributed continuous variables, while the Mann–Whitney test was used for non-normally distributed data. We employed the chi-square test for categorical data variables to compare clinical characteristics between subjects with GDM and the control group. We have conducted a Pearson correlation analysis to identify a significant correlation between maternal glycemic, lipidic, and adiponectin levels at 24–28 WG and birth and newborn weight.

Additionally, we have applied logistic regression analysis to identify possible predictors of macrosomia. We consider maternal BMI, age, and hypertensive disorders of pregnancy as potential confounding variables. In the regression model, BMI is treated as a continuous variable to assess its effect while controlling for its influence on the outcome (newborn macrosomia). Furthermore, age is included as a covariate in the logistic regression model, enabling the analysis to account for variations in age-related risks while investigating the relationship between other variables and newborn macrosomia. Hypertensive disorders are categorized as a variable (present/absent) in the regression model to adjust for their influence on the correlation between the independent variables and newborn macrosomia. A two-sided *p* < 0.05 was considered statistically significant.

## 3. Results

### 3.1. Maternal and Neonatal Demographic, Anthropometric Parameters

Table 1 presents the demographic and anthropometric parameters of the pregnant women and their newborns included in the study. We found that gestational diabetes mellitus (GDM) patients had a significantly higher incidence of a heredo-collateral history of type 2 diabetes mellitus (T2DM) (*p* < 0.0001) and a lower gestational age at delivery than the control group (*p* = 0.001).

GDM patients had a greater pre-pregnancy BMI (*p* < 0.0001), a greater BMI at 24–28 WG and at birth (*p* < 0.0001), and a lower GWG (*p* = 0.004) than the control group. Newborns from GDM mothers were heavier than newborns from control mothers (*p* = 0.01).

### 3.2. Allele and Genotype Distribution Between Newborns from the GDM and the Control Mothers Group

Table 2 shows the frequencies and distribution of alleles and genotypes in the rs266729 polymorphism in newborns from GDM and control patients. The CG genotype of rs266729 in newborns from GDM mothers is significantly higher (*p* = 0.04) than in newborns from control mothers. There were no differences between the C and G alleles of rs266729 (*p* = 0.4). The genotype distributions in GDM were out of HWE (*p* = 0.02) and in HWE in control cases (*p* = 0.40).

We did not find significant differences in the allele and genotype distributions of normoponderal and macrosomic newborns from the GDM mothers’ group compared to the control mothers’ group. The data are shown in Table 3.

### 3.3. Maternal Biochemical Parameters, Adiponectin Levels, and Newborn Anthropometric and Adiponectin Levels

In the GDM pregnant women group, the levels of adiponectin at birth and maternal fasting glucose levels at 24–28 weeks of gestation (WG) were higher in women who delivered macrosomic newborns (*p* = 0.005 and *p* < 0.0001), while maternal LDL cholesterol at 24–28 WG (*p* = 0.02) was lower compared to those who gave birth to normoponderal newborns. Data are shown in Table 4.

Adiponectin levels from the cord blood of newborns from GDM mothers were smaller than those of newborns from control mothers (19,863 ± 8013 ng/mL vs. 32,981 ± 32,187 ng/mL, *p* < 0.0001). In the newborns from the GDM mothers’ group, there were no significant differences in cord blood adiponectin levels between macrosomic and normoponderal newborns (17463 ± 7639 vs. 20506 ± 8054 ng/mL, *p* = 0.19).

**Table 4:** Characteristics of gestational diabetes mellitus (GDM) pregnant women and their newborn.

Parameters	Macrosomic Newborn *n* = 15	Normoponderal Newborn *n* = 56	*p* Value
Maternal adiponectin level at 24–28 WG, ng/dL, mean, (SD)	5976 ± 2830	5499 ± 2266	0.55
Maternal adiponectin level at birth, ng/dL, mean, (SD)	8109 ± 4531	5724 ± 2142	0.005
Maternal T-cholesterol at 24–28 WG, mg/dL, mean, (SD)	229 ± 35	241 ± 49	0.27
Maternal HDL cholesterol at 24–28 WG, mg/dL, mean, (SD)	69 ± 10	66 ± 17	0.42
Maternal LDL cholesterol at 24–28 WG, mg/dL, mean, (SD)	127 ± 23	148 ± 46	0.02
Maternal TG at 24–28 WG, mean, (SD)	273 ± 114	221 ± 76	0.08
Maternal fasting glucose level, at 24–28 WG, mg/dL, mean, (SD)	106 ± 17	93 ± 10	<0.0001
Maternal 1 h glucose level, at 24–28 WG, mg/dL, mean, (SD)	191 ± 53	172 ± 30	0.08
Maternal 2 h glucose level, at 24–28 WG, mg/dL, mean, (SD)	140 ± 52	136 ± 34	0.73
Maternal T-cholesterol at birth, mg/dL, mean, (SD)	245 ± 59	257 ± 58	0.47
Maternal HDL cholesterol at birth, mg/dL, mean, (SD)	65 ± 14	68 ± 21	0.44
Maternal LDL cholesterol at birth, mg/dL, mean, (SD)	136 ± 47	157 ± 52	0.15
Maternal TG level at birth, mg/dL, mean, (SD)	312 ± 115	292 ± 129	0.18
Newborn weight, g, mean, (SD)	4218 ± 240	3310 ± 399	<0.0001
Cord blood adiponectin, ng/dL, mean, (SD)	174637 ± 639	20506 ± 8054	0.19

Note: Data are presented as means (standard deviation). T-cholesterol = total cholesterol; HDL cholesterol = high-density lipoprotein cholesterol; LDL cholesterol = low-density lipoprotein cholesterol; TG = triglyceride; SD = standard deviation; WG = week of gestation.

In the control group, the maternal 1 h glucose levels at 24–28 WG of the pregnant women who gave birth to macrosomic newborns were lower than those of women who gave birth to normoponderal newborns. The data are shown in Table 5.

Adiponectin levels in the cord blood of macrosomic newborns from control mothers were significantly lower than those of normoponderal newborns from control mothers (23,845 ± 2575 vs. 33,314 ± 32,721 ng/mL, *p* = 0.002).

**Table 5:** Characteristics of control pregnant women and their newborns.

Parameters	Macrosomic Newborn *n* = 5	Control—Normoponderal, *n* = 137	*p* Value
Maternal adiponectin level at 24–28 WG, ng/dL, mean, (SD)	6750 ± 1260	6566 ± 2297	0.77
Maternal adiponectin level at birth, ng/dL, mean, (SD)	7629 ± 479	7338 ± 3618	0.44
Maternal T-cholesterol at 24–28 WG, mg/dL, mean, (SD)	241 ± 52	247 ± 37	0.8
Maternal HDL cholesterol at 24–28 WG, mg/dL, mean, (SD)	74 ± 12	73 ± 14	0.94
Maternal LDL cholesterol at 24–28 WG, mg/dL, mean, (SD)	139 ± 49	154 ± 36	0.38
Maternal TG at 24–28 WG, mg/dL, mean, (SD)	218 ± 121	199 ± 68	0.91
Maternal fasting glucose level, at 24–28 WG, mg/dL, mean, (SD)	77 ± 9	80 ± 5	0.25
Maternal 1 h glucose level, at 24–28 WG, mg/dL, mean, (SD)	109 ± 12	125 ± 24	0.04
Maternal 2 h glucose level, at 24–28 WG, mg/dL, mean, (SD)	96 ± 10	103 ± 18	0.23
Maternal T-cholesterol at birth mg/dL, mean, (SD)	258 ± 48	270 ± 44	0.55
Maternal HDL cholesterol at birth, mg/dL, mean, (SD)	73 ± 17	69 ± 16	0.59
Maternal LDL cholesterol at birth, mg/dL, mean, (SD)	156 ± 46	172 ± 44	0.44
Maternal TG at birth, mg/dL, mean, (SD)	283 ± 112	284 ± 89	0.89
Newborn weight, g, mean, (SD)	4486 ± 394	3303 ± 348	<0.0001
Cord blood adiponectin, ng/dL, mean, (SD)	23845 ± 2575	33314 ± 32721	0.002

Note: Data are presented as means (standard deviation). T-cholesterol = total cholesterol; HDL cholesterol = high-density lipoprotein cholesterol; LDL cholesterol = low-density lipoprotein cholesterol; TG = triglyceride; SD = standard deviation; WG = week of gestation.

In the correlation analysis of newborn weight, we found a weak positive correlation between the weight of newborns from GDM mothers and cord blood adiponectin (r = 0.262), maternal fasting glucose levels at 24–28 weeks (r = 0.288), and maternal adiponectin levels at birth (0.334). Additionally, we observed a weak negative correlation between maternal LDL-cholesterol levels at 24–28 WG and newborn weight. The data are shown in Table 6.

We found a weak negative correlation between maternal adiponectin levels at birth and newborn weight in the newborn group from the control mother group (r = 0.032).

**Table 6:** Correlation between newborn weight and cord blood adiponectin levels, maternal glycemic, lipid metabolism, and maternal adiponectin levels at 24–28 weeks and birth.

		Cord Blood Adiponectin	Maternal Adiponectin at 24–28 WG	Maternal Adiponectin Level at Birth	Maternal Fasting Glucose at 24–28 WG	Maternal 1 h Glucose Level, at 24–28 WG	Maternal 2 h Glucose Level, at 24–28 WG	Maternal T-Cholesterol at 24–28 WG	Maternal HDL Cholesterol at 24–28 WG	Maternal LDL Cholesterol at 24–28 WG	Maternal TG at 24–28 WG	Maternal T-Cholesterol at Birth	Maternal HDL Cholesterol at Birth	Maternal LDL Cholesterol at Birth	Maternal TG at Birth
Newborn weight from the GDM mothers group	r	0.262	−0.059	0.334	0.288	−0.067	−0.049	−0.114	0.161	−0.251	0.133	−0.087	−0.076	−0.169	0.074
	*p*	0.027	0.632	0.040	0.017	0.588	0.694	0.355	0.193	0.041	0.28	0.477	0.536	0.166	0.546
Newborn weight from the control mothers group	r	−0.054	−0.005	−0.181	−0.099	−0.127	−0.068	−0.031	0.005	−0.076	0.051	−0.050	0.045	−0.065	−0.002
	*p*	0.520	0.956	0.032	0.254	0.142	0.438	0.723	0.952	0.385	0.561	0.555	0.592	0.442	0.979

Note: The correlation is significant at the 0.05 level (2-tailed). Pearson correlation was used. GDM = gestational diabetes mellitus; T-cholesterol = total cholesterol; HDL cholesterol = high-density lipoprotein cholesterol; LDL cholesterol = low-density lipoprotein cholesterol; TG = triglyceride; and WG = week of gestation.

Table 7 presents the logistic regression analysis for the predictors of macrosomia. We found that the cord blood adiponectin level had an odds ratio (OR) of 6.754, the maternal fasting glucose level at 24–28 WG had an OR of 8.911, and the maternal adiponectin level at birth had an OR of 0.187 for macrosomia.

Table 8 displays the results of a multivariate logistic regression adjusted for body mass index, age, and hypertensive disorders of pregnancy concerning the predictors of macrosomia. We found that the cord blood adiponectin level, the maternal fasting glucose level at 24 to 28 weeks of gestation, and the maternal adiponectin level at birth were the strongest predictors of macrosomia in our cohort.

## 4. Discussion

In this study, we aimed to evaluate the effect of the newborn ADIPOQ rs266729 polymorphism on newborn weight status, assess the impact of maternal glycemic and lipid metabolism and maternal adiponectin levels at 24 to 28 weeks of gestation and at birth on newborn weight, and to examine the connection between cord blood adiponectin and newborn weight in a cohort of pregnant women with GDM and their newborns.

### 4.1. Maternal and Neonatal Demographic, Anthropometric Parameters

We found that gestational diabetes mellitus (GDM) patients had a significantly higher incidence of a heredo-collateral history of type 2 diabetes mellitus (T2DM) (*p* < 0.0001) and a lower gestational age at delivery compared to the control group (*p* = 0.001), which aligns with our previous findings [28] and those of Monod et al. [29]. Monod et al. [29] showed that pregnant women with first- and second-degree relatives with T2DM already had adverse glucometabolic profiles in early pregnancy.

We found that pregnant women from the GDM group had a greater pre-pregnancy body mass index (BMI) (*p* < 0.0001), a greater BMI at 24–28 weeks of gestations (WG) and birth (*p* < 0.0001), and a lower gestational weight gain (GWG) (*p* = 0.004) than the control group. We also found that, in our cohort, pregnant women from both groups had a GWG greater than the Institute of Medicine recommendation for the corresponding BMI class of our cohort of patients [30]. Song et al. [31] and ourselves, in a previous study [32], also found that the pregnant women with GDM were overweight or obese prior to pregnancy. Sun et al. [33] found that being overweight or obese before pregnancy, along with excessive weight gain during gestation, is linked to a higher risk of developing GDM and macrosomia. Previous research has indicated that being overweight or obese is a significant risk factor for GDM, primarily as a result of dysfunction in the pancreatic β-cells and the presence of insulin resistance in both skeletal muscles and adipose tissue. These changes arise from modifications in the downstream regulators of insulin signaling, mutations in the insulin receptor substrate (IRS), endoplasmic reticulum stress during insulin processing, and the influence of pro-inflammatory cytokine levels (the upregulation of IL-6 and tumor necrosis factor-alpha (TNF-alpha), along with the downregulation of IL-4 and IL-10), as well as adipokine (lower adiponectin and higher leptin) levels [34].

### 4.2. Allele and Genotype Distribution Between Newborns from the GDM Mother Group and the Control Mother Group

We found that the incidence of the CG genotype of rs266729 in newborns of GDM mothers is significantly higher (*p* = 0.04) than in the newborns of control mothers. There were no differences between the C and G alleles of rs266729 (*p* = 0.4). Additionally, we examined the allele and genotype distribution between normoponderal and macrosomic newborns from GDM and control mothers to determine whether an association exists between specific alleles or genotypes and newborn weight. We did not find significant differences in the distribution of alleles and genotypes between normoponderal and macrosomic newborns from the GDM and control mothers’ groups. Similar to our findings, Kong et al. [23] discovered that SNP rs266729 in the promoter region was not linked to birth sizes in a cohort of Korean newborns. A potential confounder in Kong et al. [23]’s work could be that the rs266729 effect on newborn weight was analyzed alongside the rs182052 impact. They did not find weight differences between newborns with the rs182052 A allele and those with the rs266729 C allele in the promoter region of the ADIPOQ gene, leading to the conclusion that rs266729 does not affect birth weight.

One explanation for the lack of association between rs266729 and newborn weight in our cohort could be the small number of patients in the GDM group, which may also contribute to being out of the HWE (*p* = 0.02). Another explanation might be that rs266729 does not play a significant role in gene expression or as a marker of a functional site in the ADIPOQ gene [35]. This indicates that factors other than SNP rs266729 could influence newborn weight.

Contrary to our findings, Saito et al. [22] found in a cohort of Japanese neonates that neonates carrying the G allele of rs266729 had significantly greater birth weights than those homozygous for the C allele. Saito et al. [22] and Kong et al. [23] found that the influence of the SNPs in the ADIPOQ gene on newborn weight may depend on circulating adiponectin.

### 4.3. Maternal Biochemical Parameters, Adiponectin Levels, and Newborn Anthropometric and Adiponectin Levels

In the GDM pregnant women group, we found that the maternal fasting glucose levels at 24–28 WG were greater (*p* < 0.0001) in the pregnant women who gave birth to macrosomic newborns than in the pregnant women who gave birth to normoponderal newborns. Also, we found a weak positive correlation between the newborn weight and the maternal fasting glucose level at 24–28 WG (r = 0.288), and in the logistic regression analysis, the maternal fasting glucose level at 24–28 WG had an OR of 8.91 for macrosomia, and an OR of 11.59 after adjusting for BMI, age, and hypertensive disorders of pregnancy. Catalano et al. [9] found that the fasting glucose level of mothers was the most significant indicator of fat mass in infants born to women with GDM. In their retrospective study involving 3211 singleton pregnant women with GDM, Wei et al. [36] showed that maternal fasting glucose at 24–28 WG was linked to increased birth weight risk and macrosomia. Like us, Wei et al. [36] did not find an association between the 1-hour and the 2-hour glucose levels at 24–28 WG and macrosomia. In their study, Sesmilo et al. [37] showed that fasting plasma glucose (FPG) in the first and second trimesters of pregnancy was significantly associated with large for gestational age (LGA). Silva et al. [38] demonstrated that the most common factors linked to neonatal macrosomia included elevated 2 h glycemia levels in the oral glucose tolerance test (OGTT), delayed treatment initiation, and fewer appointments during pregnancy. Catalano et al. [39] demonstrated in their paper that the hyperglycemia–hyperinsulinemia hypothesis (Pedersen hypothesis) is partially involved in the occurrence of macrosomia in pregnant women with GDM.

The practical implications of high maternal fasting glucose levels as a predictor of macrosomia are that healthcare providers can effectively develop strategies to improve maternal and neonatal outcomes. This should include improving maternal glucose levels through timely dietary adjustments, increased physical activity, and initiating insulin treatment when diet and exercise do not adequately enhance maternal glycemic status to lower the risk of macrosomia. By performing repeated ultrasound examinations to monitor fetal growth and planning the appropriate timing and location for delivery, healthcare providers can reduce the complications of birth associated with a macrosomic baby.

Regarding low-density lipoprotein (LDL) cholesterol, at 24–28 WG level in the pregnant GDM women group, who gave birth to macrosomic newborns, it was lower than the LDL cholesterol levels at 24–28 WG (*p* = 0.02) of pregnant women from the control group. Also, we did not find significant differences between the maternal total (T)-cholesterol, high-density lipoprotein cholesterol (HDL)-cholesterol, and triglyceride (TG) levels of mothers who gave birth to macrosomic and normoponderal newborns at 24–28 WG and at birth. Li et al. [40] found that women with GDM had higher TG levels and lower HDL cholesterol levels than those of control; they did not find any differences in maternal LDL cholesterol levels. Wang et al. [41] found that TG, T-cholesterol, and LDL cholesterol levels were higher in women with GDM than in control women, and HDL cholesterol was lower in the GDM group. Ryckman et al. [42] suggests that, regarding lipid metabolism during pregnancy in the first trimester, hypertriglyceridemia rather than hypercholesterolemia may underlie the resulting insulin resistance. The early differences in triglyceride observed between women with and without GDM may be due to a lipoprotein lipase deficiency resulting from increased fat intake and worsening insulin resistance.

Regarding the control group, the maternal 1 h glucose levels of the pregnant women who gave birth to macrosomic newborns at 24–28 WG were lower than those of women who gave birth to normoponderal newborns. We did not find differences between the maternal T-cholesterol, HDL cholesterol, LDL cholesterol, and TG levels of mothers who gave birth to macrosomic and normoponderal newborns at 24–28 WG and at birth. Sesmilo et al. [43] found that there is a positive association between free plasma glucose and LGA (*p* < 0.001) in healthy pregnant women. Ong et al. [44] found that fetal macrosomia was independently related to the mother’s fasting glucose level (OR of 2.61). Kanmaz et al. [45] found that HDL cholesterol was significantly lower, while T-cholesterol, TG, and LDL cholesterol were significantly higher in the macrosomic group (p < 0.001) of non-diabetic pregnant women. They found a significant correlation between TG levels in the second trimester of pregnancy and macrosomia. On the contrary, Retnakaran et al. [46] found that among women without GDM, maternal prepregnancy BMI, GWG up to OGTT, and leptin levels were the strongest metabolic determinants of having an LGA infant rather than glucose intolerance and lipid levels.

In the GDM pregnant women group, adiponectin levels at birth were greater in the women who gave birth to macrosomic newborns. In contrast, Horosz et al. [47] found no differences in adiponectin levels between mothers of macrosomic and non-macrosomic neonates. In one of our previous studies [28], we found that the maternal adiponectin levels at birth in the GDM group were lower than in the control group. Balachandiran et al. [19] concluded that decreased maternal adiponectin and increased insulin growth factor 1 (IGF-1) levels in the third trimester of pregnancy may have led to heightened glucose transporter protein 1 (GLUT-1) expression due to the increased activation of insulin/IGF-1 signaling in the placentas of women with GDM, which could have influenced fetal growth.

Newborns of mothers with gestational diabetes mellitus (GDM) exhibited lower levels of adiponectin in their cord blood compared to the newborns of control mothers (19863 ± 8013 vs. 32,981 ± 32187 ng/mL, *p* < 0.0001). Additionally, these levels were twice as high as the maternal adiponectin levels prior to childbirth. In the logistic regression analysis, the cord blood adiponectin level had an odds ratio (OR) of 6.754 for macrosomia and an OR of 30.31 after adjusting for BMI, age, and hypertensive disorders in pregnancy. In accordance with our findings, Cortelazzi et al. [48] and Zhang et al. [49] found that newborns of mothers with GDM had higher birth weights and lower adiponectin levels than those in the control group. They suggested that reduced cord adiponectin levels might be linked to excessive fetal adipose tissue, potentially influenced by adverse events occurring in utero. In their review, Desoye et al. [50] stated that fetal hyperglycemia and the lipogenic effects of fetal insulinemia in diabetic mothers could induce adipocyte hypertrophy. Still, they might also alter the number of adipocytes produced during the complex process of fetal adipogenesis.

Contrary to our findings, Ballesteros et al. [51] did not find differences in total adiponectin and its multimeric forms in the cord blood of offspring of GDM and control mothers. One explanation for the differing results regarding cord blood adiponectin levels in the newborns of GDM mothers compared to control mothers is that, in our study, the newborns of GDM mothers were heavier than the newborns of control mothers (3470 g vs. 3350 g, *p* = 0.01), and in the study of Ballesteros et al. [51], the weights of newborns were similar between groups (3255 g vs. 3275 g) at the same gestational age at delivery.

We found no significant differences in cord blood adiponectin levels between macrosomic and normal-weight newborns born to mothers with GDM. On the contrary, Atègbo et al. [18] found that the macrosomic offspring of GDM mothers exhibit hyperinsulinemia, low leptin, low adiponectin, low tumor necrosis factor-α (TNF-α) and IL-6, and an upregulated Th1 phenotype of T cells compared with the normoponderal newborns of control mothers. The study of Manoharan et al. [52] indicates that the newborns of mothers with GDM possess a higher ponderal index, increased fetal insulin resistance, and decreased insulin sensitivity compared to those born to control mothers. Additionally, a reduction in cord plasma adiponectin levels was observed in the GDM newborns, while the elevated leptin/adiponectin ratio showed a positive correlation with fetal insulin resistance. Cekmez et al. [53] also found that the LGA neonates had high insulin, HOMA-IR, visfatin, and apelin and low adiponectin levels. These findings suggest a link between higher insulin resistance and altered adipokine levels in the newborns of GDM mothers, which are associated with fetal adiposity. In the group of newborns of the GDM mothers, we found a weak positive correlation between adiponectin cord blood and newborn weight (r = 0.262). Similarly, Aramesh et al. [54] found a positive correlation between cord blood adiponectin and birth weight in the GDM group but not with infant length or head circumference. Chen et al. [55] also found that the newborn weight positively correlates with cord blood levels of adiponectin and prepregnancy and prepartum maternal obesity.

In the newborns from the control mothers, we found that the macrosomic newborns had lower adiponectin levels compared to normoponderal newborns. Xing et al. [56] found that the maternal and cord serum levels of leptin, the leptin/adiponectin ratio (LAR), glucose, and TG in the macrosomia group were higher than those in the control group, while the levels of HDL-C were lower. Additionally, according to Xing et al. [56], since leptin, adiponectin, and insulin are macromolecular substances, they do not pass through the placenta and cannot be directly transmitted between maternal and fetal circulation. Therefore, their respective pancreatic islets and adipose tissue produce insulin, adiponectin, and leptin in both cord and maternal blood. Mazaki-Tovi et al. [57] suggested that lower adiponectin levels in LGA newborns are related to negative feedback from adipose tissue on adiponectin production.

In conclusion, we can speculate that the lower levels of adiponectin in the cords of macrosomic newborns, compared to normoponderal newborns, are due to increased adiposity and altered adipocyte endocrine function, similar to what is seen in obese adults [58,59,60].

The clinical implication of our research is that we found no significant differences in the allele and genotype distribution of rs266729 between normoponderal and macrosomic newborns from the GDM and control mothers’ groups. Therefore, this SNP should not be considered a predictor of macrosomia. Given the limited number of patients in our pilot study, we recommend further research with larger sample size studies.

Since mothers with GDM were heavier than control mothers and fasting glucose levels at 24–28 weeks of gestation were the strongest predictors of macrosomia, healthcare providers need to advise obese young women on weight management before they become pregnant. This is essential to minimize the negative effects of obesity on both pregnant women and their children.

The findings of this study can be extended to other populations of healthy and GDM pregnant women with similar anthropometric and medical characteristics.

We acknowledge that our study has several limitations. First, we assumed that our study was a pilot study and that our sample size was limited. Second, we examined only one SNP of the ADIPOQ gene, recognizing that other ADIPOQ gene SNPs could impact newborn weight. Third, we did not assess the participants’ diets, which can significantly influence GDM occurrence and fetal weight. Lastly, we did not evaluate cord blood glucose, insulin, IR-HOMA, and lipids as newborn glycemic and lipid status indicators.

The strength of our study lies in the fact that all our patients were managed by the same team, which ensured uniformly collected data and management of the cases. We examined the effects of maternal glycemic and lipid parameters and adiponectin levels on newborn weight during the second trimester of pregnancy and at birth. We assess the relationship between newborn weight and cord blood adiponectin levels in a cohort of newborns from GDM mothers compared with a cohort of newborns from healthy mothers.

Since gestational diabetes mellitus is a multifactorial disease, future research should focus on examining the impacts of maternal metabolic factors on macrosomia in larger and more diverse cohorts. It should also study the effects of DNA methylation, histone acetylation, short-chain fatty acids, and gene expression in mothers, fetuses, and placental tissue associated with macrosomic newborns in GDM pregnancies.

## 5. Conclusions

The preliminary findings of our pilot study suggest that in the gestational diabetes mellitus group, the ADIPOQ rs266729 polymorphism in newborns is not associated with higher birth weight, the maternal fasting glucose levels between 24 and 28 WG are a predictor for macrosomia, and cord blood adiponectin levels are lower than those from control mothers. The maternal adiponectin levels in the GDM group at birth are higher in those who gave birth to macrosomic newborns and are positively correlated with the newborn weight. Considering our pilot study’s limited number of patients, we recommend further research with larger-scale studies to confirm our findings.

## Figures and Tables

**Table 1:** Demographic and anthropometric of gestational diabetes mellitus (GDM) mothers and their newborns and control cases.

Parameters	GDM Group (*n* = 71)	Control Group (*n* = 142)	*p*-Value
Maternal age at delivery, median (IQR)	33.0 (31.0–34.0)	31.0 (30.0–32.0)	0.051
Heredo-collateral history of T2DM, %	25 (35.2%)	15 (10.6%)	<0.0001
Gestation, median (IQR)	2.0 (1.0–3.0)	2.0 (1.0–3.0)	0.2
Parity, median (IQR)	2.0 (1.0–4.0)	1.0 (1.0–4.0)	0.2
Gestational age at delivery, weeks, median (IQR)	38.6 (38.2–39.3)	39.2 (38.5–39.5)	0.001
Pre-pregnancy BMI, Kg/m^2^, mean (SD)	28.1 ± 5.6	22.3 ± 3.8	<0.0001
BMI at 24–28 WG, Kg/m^2^, mean (SD)	30.9 ± 5.4	26.2 ± 3.7	<0.0001
GWG, mean (SD)	12.7 ± 7.1	15.3 ± 5.4	0.004
BMI at birth, Kg/m^2^, mean (SD)	33.1 ± 5.5	28.5 ± 3.8	<0.0001
Newborn weight, g, median (IQR)	3470 (3170–3850)	3350 (3108–3603)	0.01

Note: Data are presented as medians (standard deviation or interquartile range), counts, and percentages. T2DM = type 2 diabetes mellitus; BMI = body mass index; WG = week of gestation; GWG = gestational weight gain; IQR = interquartile range; SD = standard deviation.

**Table 2:** Comparison of newborn genotype frequencies between newborns from gestational diabetes mellitus (GDM) cases and newborns from the control group.

Parameters	GDM Group (*n* = 71) %		Control Group (*n* = 142) %		*p*-Value
rs266729					
Allele					
C	101	71.1%	212	74.6%	0.4
G	41	28.8%	72	25.3%	
Genotype					
CC	32	45.1%	81	45.1%	0.04
CG	37	52.1%	50	35.2%	
GG	2	2.8%	11	35.2%	

**Table 3:** Allele and genotype comparison between macrosomic and normoponderal newborns from gestational diabetes mellitus (GDM) and control mothers.

	Macrosomic Newborn from GDM Mothers *n* = 15	Normoponderal Newborn from GDM Mothers *n* = 56	*p* Value	Macrosomic Newborn from Control Mothers *n* = 5	Normoponderal Newborn from Control Mothers *n* = 137	*p* Value
rs266729_alele						
C	22(73.3%)	79 (70.5%)	0.82	8(80%)	204(74.4%)	>0.99
G	8 (26.6%)	33 (29.4%)		2 (20%)	70 (25.5%)	
rs266729						
CC	7 (46.7%)	25 (44.6%)	0.46	3 (60%)	78 (56.9%)	>0.99
CG	8 (53.3%)	29 (51.8%)		2 (40.0%)	48 (35.0%)	
GG	0 (0.0%)	2 (3.6%)		0 (0.0%)	11 (8.0%)	

**Table 7:** Logistic regression for the predictors for newborn macrosomia.

Parameter	*p*-Value	OR (95%CI)
Cord blood adiponectin	0.048	6.754 (0.93–44.9)
Maternal adiponectin level at 24–28 WG	0.883	1.131 (0.22–5.82)
Maternal fasting glucose level at 24–28 WG	0.011	8.911 (1.64–48.16)
Maternal 1 h glucose level, at 24–28 WG	0.717	0.755 (0.16–3.43)
Maternal 2 h glucose level, at 24–28 WG	0.880	1.140 (0.2–6.22)
Maternal adiponectin level at birth	0.038	0.187 (0.03–0.91)

**Table 8:** Results of multivariate logistic regression adjusted by body mass index (BMI), Age, and hypertensive disorders of pregnancy.

Parameter	*p*-Value	OR (95%CI)
Cord blood adiponectin	0.016	30.31 (1.81–488.39)
Maternal adiponectin level at 24–28 WG	0.443	0.405 (0.04–4.07)
Maternal fasting glucose level at 24–28 WG	0.018	11.59 (1.51–88.59)
Maternal 1 h glucose level, at 24–28 WG	0.791	0.790 (0.13–4.52)
Maternal 2 h glucose level, at 24–28 WG	0.561	1.754 (0.26–11.67)
Maternal adiponectin level at birth	0.019	0.08 (0.01–0.67)

## Data Availability

The data that support the findings of this study are available from the corresponding author C.M. upon reasonable request due to privacy and ethical reasons.
